# Epithelioma of the Tongue

**Published:** 1907-05-18

**Authors:** 


### EPITHELIOMA OF THE TONGUE.

There are few situations where a cancer is better placed for removal than in the tongue. There is not an organ of the body so frequently submitted to medical inspection, nor one the affections of which are manifest more early to the owner. And yet, despite these advantages, lingual cancer is commonly fatal.

It needs no deep philosophy to find the causes of this relative failure of treatment. The fault often lies with the patient. Through ignorance, or fear lest his suspicions be correct, an individual with an ulcer or wart on his tongue is apt to abstain from getting medical advice until compelled to do so by the progress of the disease. So, by the time the surgeon is called upon for help, it is too late. Sometimes, it is melancholy to reflect, the doctor and not the patient is responsible for this fatal delay. The writer has seen a patient with an unmistakable and advanced epithelioma of the tongue which another practitioner had been treating for more than two months with a mouth-wash.

### Predisposing Causes.

The herald of lingual cancer is superficial glossitis. And superficial glossitis is chiefly brought about by syphilis and excessive smoking. So frequent are these relations that cancer of the tongue has been described as a disease of syphilitic smokers. Professor Fournier found that among 184 cases of cancer of the mouth seen in his private practice, 155 had decided syphilitic antecedents; and Professor Poirier states that, of 32 patients operated upon by him for cancer of the tongue, 27 were syphilitic. The leading part played by smoking in the production of lingual epithelioma is too well known to need any statistical references.

Thus, an individual who has had syphilis and who smokes intemperately may be regarded as a likely subject for the development of epithelioma of the tongue; and if his tongue is very tender and sensitive with superficial glossitis he is a still more likely subject. In the presence of definite well-marked leucoplakia it may be anticipated, as Mr. Butlin has shown, that cancer will develop sooner or later in the whitened area. If there be a warty growth, or an ulcer which does not speedily heal, or a raised plaque in an area of leucoplakia, the patient may be considered to have an actual cancer in the early stage, and, of course, to require the immediate aid of surgery.

### Treatment.

In considering the treatment of cancer of the tongue it is necessary to mention the method of dealing with superficial glossitis. For there is reason to hope that, by curing superficial glossitis and preventing its return, a great deal may be done to postpone the development of cancer or to prevent its occurrence altogether. To achieve this purpose, all causes contributing to produce and sustain inflammation of the lingual mucosa must be eliminated. Smoking especially is to be interdicted. Irritating articles of diet, such as pepper, curry-powder, mustard, should be avoided; and so are undiluted spirits, as well as very hot beverages and articles of food. The teeth will require attention, for there must not be any irritation of the tongue by jagged natural teeth or ill-adapted false ones. If a small wart or chronic ulcer be present it should be excised and submitted to microscopical examination.

When well-marked leucoplakia exists, it is more difficult to decide upon the line of treatment. Probably the best course is the strictest observance of those measures which are designed to prevent glossitis, and a careful watch for any early signs of epithelioma, such as an ulcer, a papilloma, or a raised plaque. When one of these lesions occurs, the only wise course is to excise the tongue.

It is not within the scope of this article to discuss the technique of the operation, save to remark that removal of the lymphatics in the submental and submaxillary regions is a usual accessory to the primary operation; an interval of between a fortnight or three weeks is often allowed to pass between the two operations, or the whole procedure can be carried out at a single sitting.

### Prognosis.

There are three questions which a patient may be expected to ask, when advised to have his tongue removed for cancer. They are as follows:—Is the operation a dangerous one? Will it cure me? Shall I be able to speak afterwards? Unless the growth be extensive the mortality of the operation for its removal is inconsiderable, the chief cause of death being pneumonia. Shock and secondary hæmorrhage account for a few deaths. But, in careful hands and in cases which are not far advanced, there is little fear that the patient will die of the operation. In neglected cases with wide diffusion of the morbid process, the immediate dangers of operative interference are increased.

As to the likelihood of recurrence, an estimate of this can be formed only from the circumstances of each individual case. With a small and early growth near the tip of the tongue, unaccompanied by any palpable enlargement of the cervical lymphatic glands, a cure may be expected. On the other hand, if the cancer be of old standing and of wide extension, or if there be distinct enlargement of the lymphatic glands below the jaw, the prognosis will be unfavourable. But even if an operation fails to cure the patient, it may afford him great relief from suffering. For if recurrence takes place, it may appear in the neck only and not in the mouth; in which case the suffering will be much less.

In regard to a patient's powers of speech, a consideration of the nature and extent of the required operation is necessary; for the result will rest largely on the position of the growth, its duration and size. If a fairly good stump can be left, and the floor of the mouth remains mobile, the patient is nearly sure to retain the power of speaking intelligibly. He will not have normal speech; for example, he will be unable to roll an "r," but he will not be dumb. For the tongue is not the only organ involved in speech-production, notwithstanding the common superstition that removal of the tongue will make a man dumb.

